# An Fc Gamma Receptor-Mediated Upregulation of the Production of Interleukin 10 by Intravenous Immunoglobulin in Bone-Marrow-Derived Mouse Dendritic Cells Stimulated with Lipopolysaccharide *In Vitro*

**DOI:** 10.1155/2013/239320

**Published:** 2013-06-18

**Authors:** Akihiro Fujii, Yuko Kase, Chiaki Suzuki, Akihito Kamizono, Teruaki Imada

**Affiliations:** Japan Blood Products Organization, Central Research Laboratory, Protein Pharmacology Research Section, 1-5-2 Minatojima Minami-machi, Chuo-ku, Kobe, Hyogo 650-0047, Japan

## Abstract

Intravenous immunoglobulin (IVIG), a highly purified immunoglobulin fraction prepared from pooled plasma of several thousand donors, increased anti-inflammatory cytokine IL-10 production, while decreased proinflammatory cytokine IL-12p70 production in bone-marrow-derived mouse dendritic cells (BMDCs) stimulated with lipopolysaccharide (LPS). The changes of cytokine production were confirmed with the transcription levels of these cytokines. To study the mechanisms of this bidirectional effect, we investigated changes of intracellular molecules in the LPS-induced signaling pathway and observed that IVIG upregulated ERK1/2 phosphorylation while downregulated p38 MAPK phosphorylation. Using chemical inhibitors specific to protein kinases involved in activation of Fc gamma receptors (Fc*γ*Rs), which mediate IgG signals, we found that hyperphosphorylation of ERK1/2 and Syk phosphorylation occurred after stimulation of BMDC with LPS and IVIG, and the increasing effect on IL-10 production was abolished by these inhibitors. Furthermore, an antibody specific to Fc*γ*RI, one of Fc*γ*Rs involved in immune activation, inhibited IVIG-induced increases in IL-10 production, but not IL-12p70 decreases, whereas the anti-IL-10 antibody restored the decrease in IL-12p70 induced by IVIG. These findings suggest that IVIG induced the upregulation of IL-10 production through Fc*γ*RI activation, and IL-10 was indispensable to the suppressing effect of IVIG on the production of IL-12p70 in LPS-stimulated BMDC.

## 1. Introduction

Dendritic cells (DCs) are potent and specialized antigen presenting cells and important regulators of innate and adaptive immunity with the ability to induce T cell activation or its suppression. After the migration from peripheral tissues to the lymphoid organs, DCs trigger immune responses by secreting proinflammatory and anti-inflammatory cytokines to regulate T cell polarization [1, 2]. Bone-marrow-derived mouse dendritic cells (BMDCs) produce proinflammatory cytokine IL-12p70 and anti-inflammatory cytokine IL-10 when stimulated with LPS [3]. Development and proliferation of type 1 effector helper T cells (Th1) is known to be under the influence of IL-12p70. IL-12p70 activates NK and T cells and its production is inhibited by IL-10 [4, 5]. DC maturation is impaired by IL-10, and Th1-mediated immune responses are attenuated by immature DC [6, 7]. It is thought to be an important role of DC to control the balance between the development of effector and regulatory helper T cells in determination of the direction toward efficient immunosuppression and immune activation.

Intravenous immunoglobulin (IVIG) is a highly purified immunoglobulin (IgG) fraction prepared from pooled plasma of several thousand donors and, based on its immunoregulatory role, is widely applied for the therapy of inflammatory and autoimmune diseases [8]. IgG consists of two major functional regions, Fab and Fc. The Fab region is mainly responsible for antigen binding including the blockade of certain receptors and neutralization of bacterial toxins, autoantibodies, or cytokines. The Fc region couples the antibody to IgG receptors, named Fc gamma receptors (Fc*γ*Rs), of the innate immune effector cells, such as neutrophils, mast cells, macrophages, and dendritic cells [8]. Although the precise mechanisms of the regulatory functions of IVIG in the immune system have not been cleared yet, several mechanisms of action have been proposed, such as the interaction of an anti-idiotypic antibody, modulation of cytokine production, inhibition of T-cell proliferation, and modulation of serum complement action on target cells [9–11].

Bayry et al. reported an interesting bidirectional effect of IVIG [12]. They showed that IVIG inhibited the production of IL-12p70 and stimulated the secretion of IL-10 in LPS-activated human monocyte-derived dendritic cells (mo-DC). As a consequence, in the presence of IVIG during mo-DC maturation or developmental stages, the T-cell stimulatory activity of mo-DC is impaired and its T-cell suppressive function appears [12]. The mechanisms of this bidirectional effect of IVIG on cytokine production are not clear. To understand the immunoregulatory function of IVIG more clearly, we focused on the relationship between this modulatory effect of IVIG on cytokine production and its actions on the signaling pathway in mouse BMDC stimulated with LPS. 

LPS signal is mediated by the transmembrane receptor, Toll-like receptor 4 (TLR4), for the induction of cytokine production in immune cells [13]. The LPS-triggered TLR4 activation induces the phosphorylation of intracellular signaling proteins including transcription factor NF*κ*-B and members of the extracellular signal-regulated kinases (ERKs) family, ERK1/2, p38 MAPK, and c-Jun N-terminal kinase (JNK). Activation of TLR4 is necessary for the maturation and functional responses of BMDC including cytokine production, phagocytosis, antigen presentation, and T-cell activation/suppression [14, 15]. Because IVIG contains a wide range of antibodies that harbor a broad repertoire of antigen binding variable regions present in normal serum, an LPS-binding fraction in IVIG is a possible factor that blocks TLR4 signaling and reduces cytokine production in DC stimulated with LPS [16]. However, it is difficult to explain that only an inhibition of TLR4 signaling results in the upregulation of cytokine production in these LPS-stimulated cells. 

BMDCs express two general classes of Fc*γ*Rs [17]. One is activation receptors, the high affinity receptor Fc*γ*RI (CD64) and the low affinity receptor Fc*γ*RIII (CD16), which mediate activating functions in immune responses. The *α*-chain of these two activation receptors associates with the cell-signaling common *γ*-chain (FcR*γ*) subunit containing the immunoreceptor tyrosine-based activation motif (ITAM), and ITAM is indispensable in the immune responses after receptor activation. The other is the inhibitory receptor Fc*γ*RIIB (CD32B) containing the immunoreceptor tyrosine-based inhibitory motif (ITIM) within the sequence and it has been shown to be critical for its inhibitory function [17]. In the case of signal transduction by Fc*γ*R after its engagement with immune complexes, the activation of several molecules in the signaling cascade of ITAM including ERK family kinases occurs and this is an important step for immune responses mediated by Fc*γ*R activation [18, 19]. The molecular targets of IVIG in immune modulation are not clear; however, activation and inhibitory Fc*γ*Rs are promising candidates through which IVIG triggers receptor functions and modulates immune responses. 

 To investigate the mechanisms of modulatory effect of IVIG on the cytokine production, we hypothesized that IVIG could affect the signaling pathway of Fc*γ*R as well as TLR-4 signal transduction in LPS-stimulated BMDC, which may affect cytokine production in these cells. In this study, we investigated changes in Fc*γ*R-mediated signaling molecules and cytokine production when cells were activated by LPS in the presence of IVIG and found that Fc*γ*RI activation was necessary to enhance IL-10 production in LPS-stimulated BMDC by IVIG.

## 2. Materials and Methods

### 2.1. Reagents and Antibodies

Venoglobulin-IH (Japan Blood Products Organization, Tokyo, Japan), which is used as an intravenous immunoglobulin (IVIG), was dialyzed against phosphate-buffered saline (PBS) at 4°C for one day and filtered through a 0.22 *μ*m membrane (Millipore, Temecula, CA). Lipopolysaccharide (LPS) from *Escherichia coli* serotype O55:B5 and penicillin/streptomycin were purchased from Sigma (St. Louis, MO). LPS was used at a concentration of 500 ng/mL or 1 *μ*g/mL. Fetal calf serum (FCS) was obtained from Gibco (Rockville, MD). Recombinant murine granulocyte-macrophage colony-stimulating factor (rmGM-CSF) was purchased from PeproTech (Rocky Hill, NJ). The culture medium was RPMI1640 supplemented with penicillin (100 u/mL), streptomycin (100 *μ*g/mL), and 10% heat-inactivated FCS. Human pooled plasma was prepared by Japan Blood Products Organization and used after heat inactivation. Protein tyrosine kinase inhibitors, piceatannol for Syk and PP2 for Hck/Lyn, were purchased from Wako (Osaka, Japan) and Enzo (Plymouth Meeting, PA), respectively [20–22].

For immunoblotting, monoclonal rabbit antinuclear factor kappa (NF*κ*-B) p65 (C22B4), monoclonal rabbit anti-phospho-NF*κ*-B p65 (Ser536, 93H1), polyclonal rabbit anti-p38, monoclonal mouse anti-phospho-p38 (Thr180/Tyr182, 28B10), monoclonal rabbit antiextracellular signal-regulated kinase 1/2 (ERK1/2, 137F5), monoclonal rabbit anti-phospho-ERK1/2 (Thr202/Tyr204, D13.14.4E), monoclonal rabbit anti-c-Jun N-terminal kinase (JNK, 56G8), monoclonal mouse anti-phospho-JNK (Thr183/Tyr185, G9), and polyclonal rabbit anti-phospho-Syk (Tyr525/526) were purchased from Cell Signaling Technology (Danvers, MA) and were used for 1,000 times in dilution. Monoclonal mouse anti-Syk (SYK-01, 1 : 1,000) was from EXBIO (Vestec u Prahy, Czech). Horse-radish-peroxidase- (HRP-) conjugated, monoclonal mouse anti-phosphotyrosine antibody (4G10 Platinum, 1 : 1,000) was from Millipore. HRP-conjugated goat anti-mouse IgG and HRP-conjugated goat anti-rabbit IgG (number 7074, 1 : 1,000) were purchased from DAKO (Glostrup, Denmark) and Cell Signaling Technology, respectively. All other materials used in this study were of reagent grade. 

### 2.2. Mice

C57BL/6 female mice were purchased from Charles River Laboratories (Kanagawa, Japan) and were maintained under specific pathogen-free conditions in accordance with the ethics and safety guidelines for animal experiments of Japan Blood Products Organization.

### 2.3. Preparation and Culture of BMDC

Methods for the preparation of BMDC were performed according to Lutz et al. with some modifications [23]. Briefly, on day 0, bone marrow cells prepared from the femurs and tibiae of 7–9-week-old female mice were adjusted to 2 × 10^5^/mL in 10 mL culture medium containing rmGM-CSF (20 ng/mL) and were maintained in 100 mm dishes (Asahi Glass, Tokyo, Japan) at 37°C in a humidified incubator with 5% CO_2_. On day 2, another 10 mL of culture medium supplemented with rmGM-CSF was added to these plates. On day 4 or 5, half of the medium was centrifuged and cells were suspended in 10 mL fresh culture medium containing rmGM-CSF and were returned to the original plates. On day 7 or 8, cells were collected and used as BMDC. At this point, over 90% of CD11c expression was confirmed by cell surface maker analysis using an FC500 flow cytometer (Beckman-Coulter, Miami, FL). 

### 2.4. Cytokine Production and Treatment with the Anti-Fc*γ*RI Antibody or Anti-IL-10 Antibody

Cells (5 × 10^5^/mL) were cultured in 48 well plates (0.5 mL/well, Asahi Glass) with or without LPS (500 ng/mL) for 6 h and 18 h. IVIG was added simultaneously at concentrations of 2.5, 5, and 10 mg/mL according to our previous study with some modifications [24]. Cell culture supernatants were assayed for IL-10 and IL-12p70 concentrations by an enzyme-linked immunosorbent assay (ELISA) using the Quantikine mouse IL-10 immunoassay and mouse IL-12p70 immunoassay (R&D Systems, Minneapolis, MN, USA), respectively, according to manufacturer's procedures. In our study, without LPS stimulation, IL-10 and IL-12p70 concentrations in the culture medium of cells were under the lower limits of the standard ELISA curves (lower limit values of standard curves of ELISA were 15.6 pg/mL for IL-10 and 7.8 pg/mL for IL-12p70). IVIG (2.5–10 mg/mL) showed no production of either cytokine in cells without LPS stimulation. IVIG did not inhibit the cell growth *in vitro*. 

 To test whether Fc*γ*RI was involved in the increase in IL-10 by IVIG, the rat monoclonal anti-mouse Fc*γ*RI/CD64 antibody (290322, R&D systems, Minneapolis, MN) and a matched control antibody (rat IgG_2A_, R&D systems) at a concentration of 1 *μ*g/mL were added to the culture medium of cells in the presence or absence of IVIG (10 mg/mL) and were stimulated with LPS (500 ng/mL) for 18 h. To study the effects of the anti-IL-10 antibody on the production of IL-12p70, cells were stimulated with LPS (1 *μ*g/mL) in the presence or absence of IVIG (5 mg/mL) and the rat monoclonal ant-mouse IL-10 (1 *μ*g/mL, JES5-25A, Southern Biotech, Birmingham, AL) or a matched control antibody (rat IgG_1_, Southern Biotech) for 18 h. Supernatants were collected and cytokine production was assayed in the same manner as that described earlier.

### 2.5. Effects of Protein Tyrosine Kinase Inhibitors on IL-10 Production

Cells (5 × 10^5^/mL) were stimulated with LPS (500 ng/mL) in the presence or absence of IVIG (10 mg/mL) and protein tyrosine kinase inhibitors for 18 h. Cytokine production in the culture supernatant was measured as described earlier. The concentrations of the inhibitors used were 5 *μ*M for piceatannol and 0.05 *μ*M for PP2, respectively. These concentrations effectively inhibited the phosphorylation of the respective kinase [20–22].

### 2.6. Quantitative-PCR of IL-10, IL-12a, and IL-12b mRNAs

Cells (5 × 10^5^/mL) were stimulated with or without LPS (500 ng/mL) in the presence or absence of IVIG in 48 well plates for 6 h and 18 h. The expressions of IL-10 mRNA and IL-12a and IL-12b mRNA, which codes the IL-12p35 and IL-12p40 subunit of IL-12p70, respectively, were measured by a quantitative reverse transcription-polymerase chain reaction (RT-PCR) using a 7500 Real-Time PCR System (Applied Biosystems, Foster City, CA). Briefly, total RNA was extracted from cells using the PureLink RNA Mini kit and Turbo DNA-free kit (Life Technologies, Carlsbad, CA) and cDNA was constructed by the High Capacity RNA-to cDNA kit (Applied Biosystems). Real-time PCR was subsequently performed using TaqMan Gene Expression Assays (Applied Biosystems), sequence specific primers, and probes according to the manufacturer's protocol. The expression of each mRNA was standardized to GAPDH mRNA, and they were expressed as ratios to the mean value for cells without stimulation. Each mRNA expression was analyzed by the ΔΔCt method as per the manufacturer's instructions [25]. The following sequence specific primers and probes were used for real-time PCR: IL-10, Mm01288386_m1; IL-12a, Mm00434169_m1; IL-12b, Mm01288993_m1; GAPDH, TaqMan Rodent GAPDH control reagents (Applied Biosystems). 

### 2.7. Immunoblot and Immunoprecipitation

Immunoblot assays were performed as described next. 

Cells (0.5–1 × 10^6^/mL) were treated with LPS (500 ng/mL) with or without IVIG (10 mg/mL) at 37°C for 15 min. When protein tyrosine kinase inhibitors were used in the assay, they were added to the culture medium at concentrations of 0.05 or 0.1 *μ*M for PP2 and 5 or 10 *μ*M for piceatannol. Reactions were stopped by rapid cooling on ice and cells were washed with ice-cold PBS once. Cells were lysed with lysis buffer containing 50 mM Tris-HCl, 1% NP40, protease inhibitors cocktail, Complete (Roche Diagnostic, Basel, Switzerland), 1 mM *β*-glycerophosphoric acid, 1 mM Na_3_VO_4_, and 5 mM NaF and were centrifuged at 12,000 ×g at 4°C for 15 min. Supernatants were collected and used as cell lysates. The protein concentrations of supernatants were measured by the BCA protein assay kit (Pierce, Rockford, IL). 

Cell lysates (25 or 50 *μ*g protein) were separated on 10% Nu-PAGE gels (Life Technologies) and electrotransferred onto nitrocellulose membranes using the iBlot transfer system (Life Technologies). After the transfer, membranes were blocked with 5% skim milk in PBS containing 0.1% Tween 20 (PBS-T) for 1 h at room temperature. After blocking, membranes were reacted with the primary antibodies described earlier at 4°C overnight. After washing with PBS-T, membranes were treated with HRP-conjugated secondary antibodies for 1-2 h at room temperature. Blots were developed with enhanced chemiluminescence reagents (Immobilon Western Chemiluminescent HRP Substrate kit, Millipore). Chemiluminescence signals were imaged using a Molecular Imager Pharos FX (Bio-Rad, Hercules, CA). 

To detect Syk phosphorylation after stimulation with LPS for 15 min with or without IVIG, cell lysates (100 *μ*g protein) were incubated with the anti-Syk antibody (1 *μ*g) and precipitated by Protein A/G (Santa Cruz, Santa Cruz, CA) at 4°C for one day. Immunoprecipitates were analyzed by immunoblotting with the HRP-conjugated, anti-phosphotyrosine antibody (4G10), anti-Phospho-Syk antibody, or anti-Syk antibody, in the same manner as that described earlier. 

### 2.8. Statistical Analysis

Results were expressed as mean values and standard error of the mean (SEM, *n* = 3). Data were analyzed by Student's *t*-test (for comparison between two group) or Dunnett's multiple comparison test (for multiple group) using the SAS system (ver. 9.1.3, SAS Institute, Cary, NC). Differences with *P* values less than 0.05 were considered to be significant. Results were representative of two or three independent experiments.

## 3. Results

### 3.1. IVIG Increased IL-10 Production Whereas Decreased IL-12p70 Production in BMDC Stimulated with LPS

**Figure 1:**
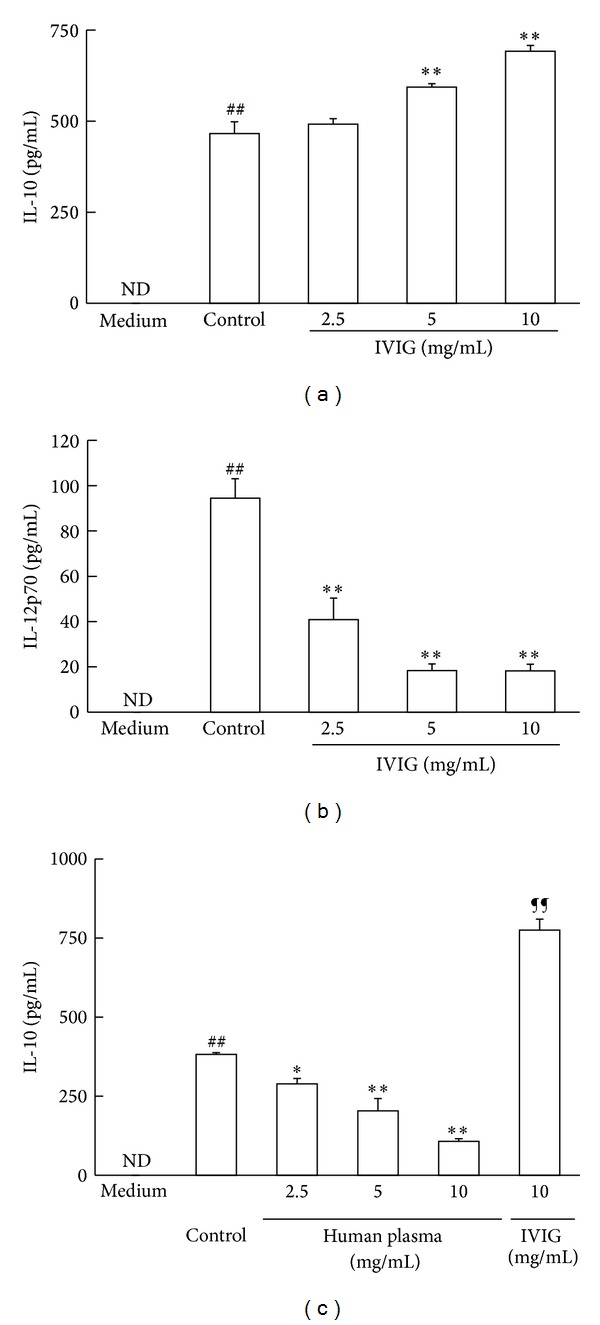
IVIG increased IL-10 production whereas decreased IL-12p70 production in BMDC stimulated with LPS. Cells were stimulated with LPS (1 *μ*g/mL) in the presence of IVIG or human pooled plasma for 18 h. Cytokine concentrations in the culture medium were determined using respective ELISA kits. (a) Production of IL-10; (b) production of IL-12p70; (c) production of IL-10 in the presence of human pooled plasma. Results were expressed as mean ± SEM (*n* = 3). ^##^ *P* < 0.01, significantly different from the medium alone (without LPS stimulation, Student's *t*-test); *P < 0.05, **P < 0.01, significantly different from the Control (LPS stimulation without IVIG, Dunnett's multiple comparison test); ^¶¶^ *P* < 0.01, significantly different from the Control, Student's *t*-test; ND, IL-10 and IL-12p70 concentrations were under the lower limit values of the standard ELISA curves. At least three independent experiments were conducted and representative results were shown.

First, we evaluated IL-10 and IL-12p70 production from BMDC stimulated with LPS and the effects of IVIG on the production of these two cytokines. After the stimulation of cells with LPS for 18 h, both IL-10 and IL-12p70 productions were clearly detected in the culture medium (Figure 1). The addition of IVIG concentration dependently increased IL-10 production which was statistically significant at concentrations of 5 and 10 mg/mL (Figure 1(a)). On the other hand, a clear decrease in IL-12p70 production was observed in the culture medium 18 h after stimulation in the presence of 2.5, 5, and 10 mg/mL IVIG (Figure 1(b)). The similar effects of IVIG were observed in cells 6 h after stimulation with LPS (Supplement data, Figure S1 available online at http://dx.doi.org/10.1155/2013/239320). These observed effects of IVIG in mouse BMDC stimulated with LPS were similar to those reported using human blood DC (12). To confirm whether another human protein had the same effect, we tested the effects of human pooled plasma on IL-10 production in BMDC stimulated with LPS. When human pooled plasma (2.5 to 10 mg/mL) was added to the culture medium of BMDC stimulated with LPS, a clear decrease in IL-10 production was observed in a concentration-dependent manner (Figure 1(c)). This indicated that human pooled plasma contained unknown factors that inhibited IL-10 production in LPS-stimulated BMDC and a xenobiotic protein exerted various influences on cytokine production in these cells. 

### 3.2. IVIG Increased IL-10 mRNA Transcription Whereas Decreased IL-12 mRNA Transcription in BMDC Stimulated with LPS

**Figure 2:**
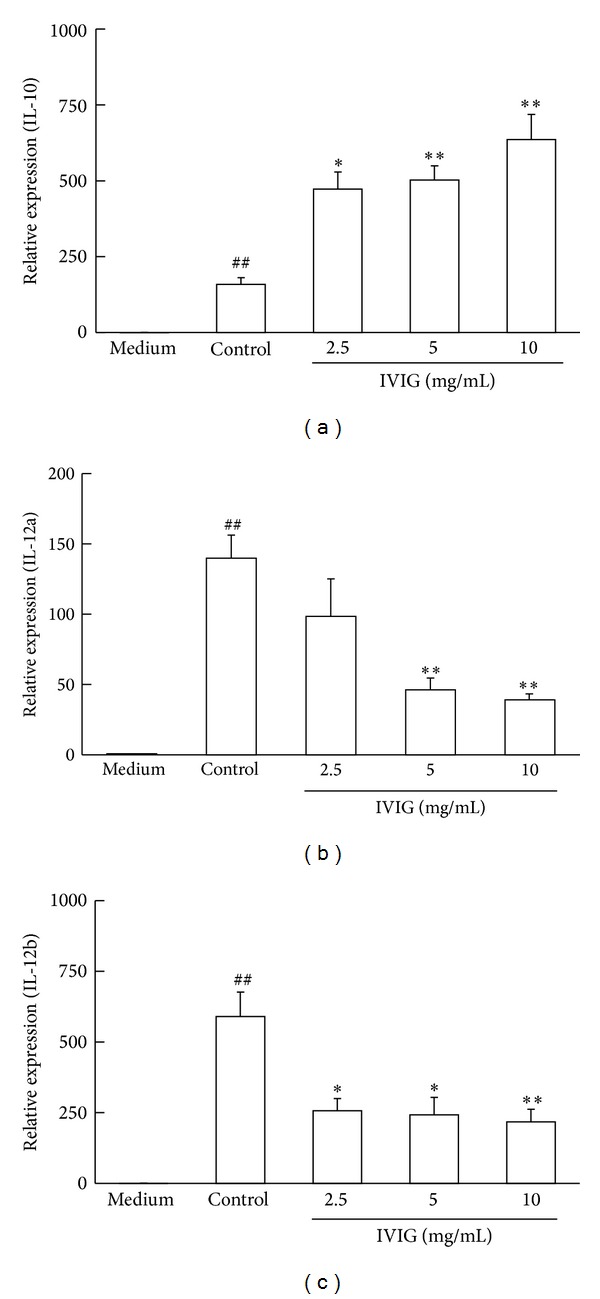
IVIG increased IL-10 mRNA transcription whereas decreased IL-12 mRNA transcription in BMDC stimulated with LPS. Cells were stimulated with LPS (1 *μ*g/mL) in the presence of IVIG for 6 h. The expressions of mRNA for IL-10, IL-12a, and IL-12b were determined by real-time quantitative RT-PCR. Results were expressed as mean ± SEM (*n* = 3). ^##^ *P* < 0.01, significantly different from the medium alone (without LPS stimulation, Student's *t*-test); *P < 0.05, **P < 0.01, significantly different from the Control (LPS stimulation without IVIG, Dunnett's multiple comparison test). At least three independent experiments were conducted and representative results were shown. (a) Expression of IL-10; (b) expression of IL-12a; (c) expression of IL-12b.

To investigate the effects of IVIG on the transcription levels of these cytokines, we next measured the mRNA expression of IL-10 as well as IL-12a and IL-12b, which codes the IL-12p35 and IL-12p40 subunits of IL-12p70, respectively, in cells stimulated with LPS. The expression of all three mRNAs was seen 6 h after the LPS treatment (Figure 2). IVIG at concentrations of 2.5, 5, and 10 mg/mL significantly increased the IL-10 mRNA expression 6 h after LPS treatment in a concentration-dependent manner (Figure 2(a)). On the other hand, 5 and 10 mg/mL IVIG clearly decreased IL-12a mRNA and 2.5, 5, and 10 mg/mL IVIG suppressed IL-12b expression in cells 6 h after stimulation (Figures 2(b) and 2(c)). The similar effects of IVIG on the expression of these three mRNA were observed 18 h after stimulation with LPS (Supplement data, Figure S2). These results suggest that IVIG affects the production and expression of both IL-10 and IL-12p70 in BMDC stimulated with LPS.

### 3.3. IVIG Affected Signaling Molecules in TLR4 Signaling Cascade of LPS-Stimulated BMDC 

**Figure 3:**
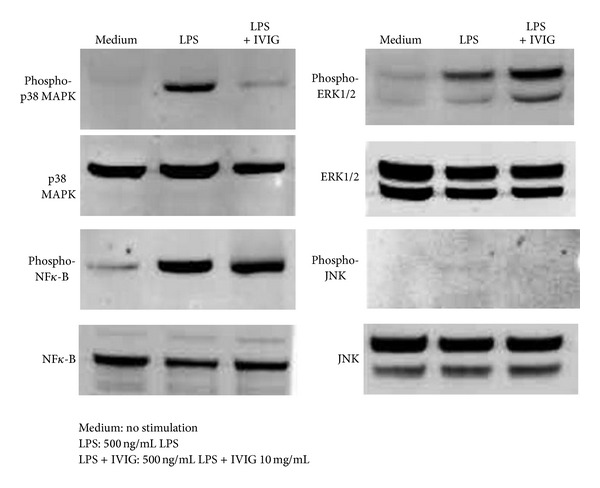
IVIG induced hyperphosphorylation of ERK1/2 and downregulated p38 MAPK phosphorylation in BMDC stimulated with LPS. Cells were stimulated with LPS (500 ng/mL) in the presence or absence of  IVIG (10 mg/mL) for 15 min. Cell lysates were incubated with specific antibodies against NF*κ*-B, ERK1/2, p38 MAPK, and JNK and their phosphorylated forms and were analyzed by respective immunoblottings.

IL-10 and IL-12p70 production in cells stimulated with LPS is mediated by activation of  Toll-like receptor 4 (TLR4) on the cell membrane [13–15]. TLR4 signal induces the phosphorylation of NF*κ*-B and the ERK family members, ERK1/2, p38 MAPK, and JNK. To investigate effects of IVIG on the LPS-TLR4 signal transduction, the phosphorylation of these proteins after the stimulation of cells with LPS was evaluated using specific antibodies by immunoblotting. The clear phosphorylation of NF*κ*-B, ERK1/2, and p38 MAPK and a very weak JNK phosphorylation signal were observed in the cell lysates 15 min after stimulation with LPS (Figure 3). IVIG decreased phosphorylation of p38 MAPK in cells stimulated with LPS. IVIG (10 mg/mL) showed no clear influence on NF*κ*-B phosphorylation. We could not detect any significant change in JNK phosphorylation by IVIG. On the other hand, an increase in ERK1/2 phosphorylation was detected in the same cell lysates (Figure 3). Although ERK1/2 hyperphosphorylation occurred by the treatment, these results suggest that IVIG at least inhibited activation of one signaling protein in TLR4 signaling cascade in BMDC stimulated with LPS. 

### 3.4. IVIG Induced Syk Phosphorylation in BMDC Stimulated with LPS

**Figure 4:**
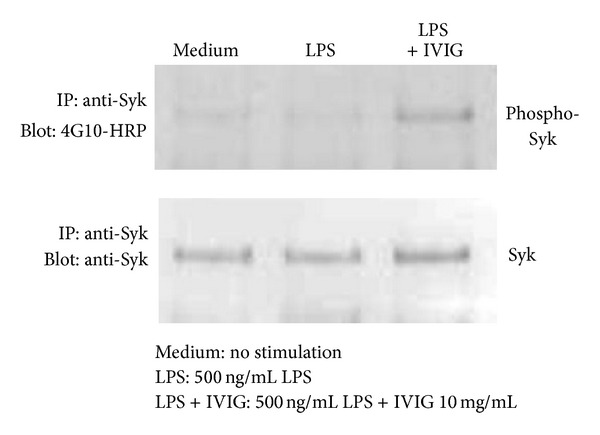
IVIG induced Syk tyrosine phosphorylation in BMDC stimulated with LPS. Cells were stimulated with LPS (500 ng/mL) in the presence or absence of IVIG (10 mg/mL) for 15 min. Cell lysates were incubated with the anti-Syk antibody and precipitated by Protein A/G. Immunoprecipitates were analyzed by immunoblotting with HRP-conjugated, anti-phosphotyrosine antibody (4G10) or the anti-Syk antibody.

As p38 MAPK and JNK activations are reported to be required for IL-12p70 production in DC [26, 27], the fact that IVIG inhibits p38 MAPK phosphorylation could explain the decrease in IL-12p70 production in BMDC stimulated with LPS and IVIG. In contrast, ERK1/2 phosphorylation is reported to be necessary for the induction of IL-10 [28–30]. As IVIG treatment induced an increase in ERK1/2 phosphorylation, we thought that hyperphosphorylation of ERK1/2 could contribute to an increase in IL-10 production by IVIG. IgG exerts its effects through Fc*γ*Rs on the cell membrane [8, 10]. We then hypothesized that when BMDCs are stimulated with LPS in the presence of IVIG, activating signals from Fc*γ*R are initiated and these result in ERK1/2 hyperphosphorylation. Therefore, we examined the effects of IVIG on phosphorylation of the Syk protein, which is required for the activation of high affinity Fc*γ*RI and low affinity Fc*γ*RIII, after the stimulation of cells with LPS [31, 32]. Analysis by the immunoprecipitation of cell lysates with the anti-Syk antibody and HRP-conjugated phosphotyrosine specific 4G10 antibody revealed that a clear increase in Syk phosphorylation was detected upon stimulation in DC stimulated with LPS and IVIG (Figure 4). In contrast, no increase in Syk phosphorylation was observed in cells stimulated with LPS alone (Figure 4). This suggests that IVIG triggers Fc*γ*R activation of BMDC when these cells are stimulated with LPS. 

### 3.5. PP2 and Piceatannol Inhibited the Enhancement of IL-10 Production Induced by IVIG

**Figure 5:**
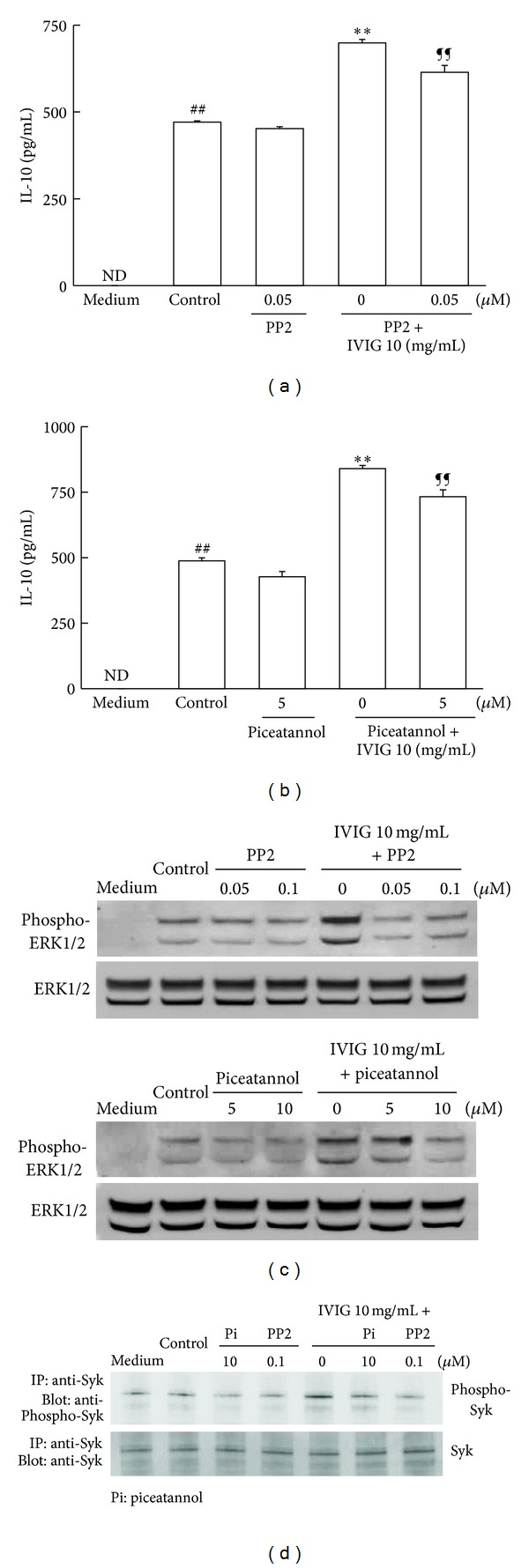
Protein tyrosine kinase inhibitors PP2 and piceatannol affected the enhancing effect of IVIG on IL-10 production in BMDC stimulated with LPS. Cells (5 × 10^5^/mL) were stimulated with LPS (500 ng/mL) in the presence or absence of IVIG (10 mg/mL) and/or protein tyrosine kinase inhibitors for 18 h. IL-10 production in the culture supernatant was measured using an ELISA kit and results were expressed as mean ± SEM (*n* = 3). ^##^ *P* < 0.01, significantly different from the medium alone (without LPS stimulation, Student's *t*-test); **P < 0.01, significantly different from the Control (LPS stimulation without IVIG, Dunnett's multiple comparison test); ^¶¶^ *P* < 0.01, significantly different from LPS stimulation with IVIG, Student's *t*-test. ND, IL-10 concentrations were under the lower limit values of the standard ELISA curves. The enhancing effect of IVIG on IL-10 production was abolished by (a) the Hck/Lyn inhibitor PP2 and (b) Syk inhibitor piceatannol. Three independent experiments were conducted and representative results were shown. The tyrosine phosphorylation of ERK1/2 and Syk in cells was detected by immunoblotting as described in the Materials and Methods. PP2 and piceatannol inhibited ERK1/2 and Syk phosphorylation by IVIG in mouse BMDC stimulated with LPS. (c) ERK1/2; (d) Syk.

The activation of Fc*γ*R is mediated by several protein tyrosine kinases. Of these kinases, the Src family kinases, Hck/Lyn, are essential for tyrosine phosphorylation in an ITAM motif of FcR*γ* subunit of Fc*γ*RI and Fc*γ*RIII, and ITAM-phosphorylated FcR*γ* activation is required for Syk activation [31–34]. Therefore, to confirm that IVIG-induced Fc*γ*R activation is involved in the enhancing effect on IL-10 production in BMDC stimulated with LPS, we tested whether the inhibition of these protein tyrosine kinases could affect the upregulation of IL-10 production by IVIG. First, the Hck/Lyn inhibitor, PP2, was added to the culture and the production of IL-10 was assessed. Treatment with PP2 at 0.05 *μ*M reduced the increase in IL-10 production by IVIG (Figure 5(a)). Next, to investigate the effects of the Syk inhibitor, piceatannol, cells were cultured with IVIG and stimulated with LPS in the presence of piceatannol (5 *μ*M) and IL-10 production was measured. The addition of piceatannol diminished the enhancing effect of IVIG on IL-10 increase (Figure 5(b)). Analysis by immunoblotting and immunoprecipitation confirmed that PP2 and piceatannol inhibited the phosphorylation of ERK1/2 and Syk induced by IVIG in cells treated with LPS (Figures 5(c) and 5(d)). These results suggest that Fc*γ*R activation induced by IVIG is necessary to increase IL-10 production in BMDC stimulated with LPS. 

### 3.6. Fc*γ*RI Was Required for the IL-10 Production Induced by IVIG

**Figure 6:**
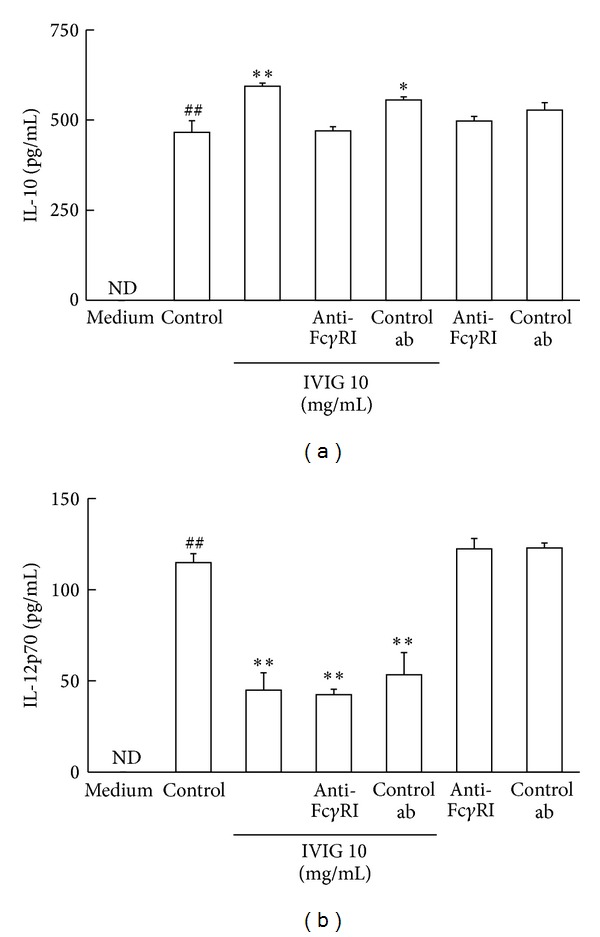
Anti-Fc*γ*RI antibody affects the cytokine productions in the presence of IVIG in BMDC stimulated with LPS. The anti-mouse Fc*γ*RI antibody and a matched control antibody were added to the cells in the presence or absence of IVIG (10 mg/mL) and cells were stimulated with LPS (500 ng/mL) for 18 h. Productions of cytokines in the culture supernatant were measured using respective ELISA kits and results were expressed as mean ± SEM (*n* = 3). ^##^ *P* < 0.01, significantly different from the medium alone (without LPS stimulation, Student's *t*-test); *P < 0.05,  **P < 0.01, significantly different from the Control (LPS stimulation without IVIG, Dunnett's multiple comparison test). ND, Cytokine concentrations were under the lower limit values of the standard ELISA curves. Three independent experiments were conducted and representative results were shown. (a) IL-10; (b) IL-12p70.

To study the role of Fc*γ*R activation in the modulatory effects of IVIG described earlier more clearly, we evaluated the effect of anti-Fc*γ*RI antibody on IL-10 and IL-12p70 production in BMDC treated with LPS and IVIG. In the presence of anti-Fc*γ*RI antibody, increment of IVIG-induced IL-10 production was clearly negated. Control antibody had no effect. Both antibodies had no influence on IL-10 production in cells stimulated with LPS and the control antibody also had not affect the IL-10 production induced by IVIG (Figure 6(a)). On the other hand, the suppressing effect of IVIG on IL-12p70 production did not change in the presence of the anti-Fc*γ*RI antibody in the assay (Figure 6(b)). These findings suggest that activation of Fc*γ*RI is necessary for IVIG to enhance the production of IL-10 but is not required for the suppression of IL-12p70 production induced by IVIG in LPS-stimulated BMDC. 

### 3.7. Suppressive Effect of IVIG on the IL-12p70 Production Was Inhibited by Anti-IL-10 Antibody

**Figure 7:**
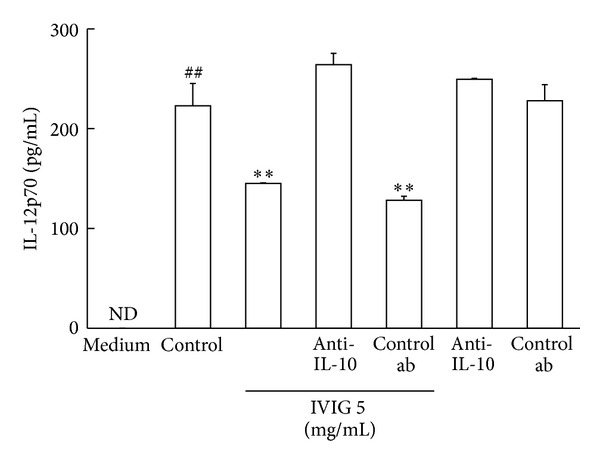
Anti-IL-10 antibody abolished the effect of  IVIG on the suppression of IL-12p70 production in BMDC stimulated with LPS. Cells were stimulated with LPS (1 *μ*g/mL) in the presence or absence of  IVIG (5 mg/mL) and the anti-IL-10 or its control antibody for 18 h. IL-12p70 production in the culture supernatant was measured using the ELISA kit. Results were expressed as mean ± SEM (*n* = 3). ^##^ *P* < 0.01, significantly different from the medium alone (without LPS stimulation, Student's *t*-test); **P < 0.01, significantly different from the Control (LPS stimulation without IVIG, Dunnett's multiple comparison test). ND, cytokine concentrations were under the lower limit values of the standard ELISA curves. Three independent experiments were conducted and representative results were shown.

It has been reported that IL-12p70 production in BMDC stimulated with LPS is regulated by IL-10 [5]. We thought that IL-10 in the culture medium could affect and decrease IL-12p70 production. Thus, we cultured the cells with LPS in the presence of IVIG and an anti-IL-10 antibody and measured IL-12p70 production in the medium. The addition of the anti-IL-10 antibody, but not its control antibody, clearly negated the effect of IVIG on IL-12p70 production. Both antibodies did not show clear effect on IL-12p70 production from the cells stimulated with LPS alone, and the control antibody also had no influence on the suppressing effect of IVIG on the production of IL-12p70 (Figure 7). These results indicate that IL-10 in the culture medium is required for the suppressing effect of IVIG on the production of IL-12p70 in BMDC stimulated with LPS.

## 4. Discussion

IVIG shows immunomodulatory effects in inflammatory and autoimmune diseases [8, 9]. 

Although the direct effects of IVIG such as on the cytokine production in peripheral blood mononuclear cells (PBMCs) induced by bacterial components, the proliferation of and cytokine production in T-cells, and TLR-mediated B-cell responses have been reported previously [24, 35–37], the molecular mechanisms of immunomodulation by IVIG are still under extensive research. To investigate the molecular targets of IVIG in the modulation of cytokine productions, we used mouse BMDC stimulated with LPS and observed that IVIG had an enhancing effect on the production of the anti-inflammatory cytokine IL-10 and, at a same time, a suppressing effect on the proinflammatory cytokine IL-12p70 production, similar to human mo-DC as reported by Bayry et al. [12]. We also confirmed that these modulations of cytokine production by IVIG occurred at the transcription level of each cytokine gene. In addition, we found the upregulation of ERK1/2 phosphorylation as well as the downregulation of p38 MAPK phosphorylation after LPS stimulation in the presence of the drug. The results of immune blotting were out of our expectation, as we hypothesized that neutralization by IVIG would inhibit LPS-induced TLR4 signaling. The exact reasons of the unexpected results were not clear, but we could not deny the possibility of neutralization by IVIG because we thought that our experimental conditions might not be suitable to detect the clear changes in NF-*κ*B phosphorylation and the phosphorylation signal of JNK was very faint and hard to detect the reduction of phosphorylation by IVIG. It was also reasonable to think that ERK1/2 phosphorylation was reduced by neutralization, but we thought that the effect of activated Fc*γ*Rs would exceed that of neutralization as discussed later. 

Several studies have reported that ERK1/2 phosphorylation is necessary for the production of IL-10 by DC after some ligand stimulations [28–30]. The fact that the upregulation of ERK1/2 phosphorylation of BMDC stimulated with LPS in the presence of IVIG correlates well with the increase in IL-10 production under the same culture conditions. It seems that IVIG exerts its action through a different pathway including ERK1/2 phosphorylation other than LPS-TLR4 signaling. Fc*γ*RI and Fc*γ*RIII are members of IgG receptor, named Fc*γ*R, which mediate activating functions in immune responses. Fc*γ*R activation is mediated by tyrosine phosphorylation of the ITAM motif in the FcR*γ* subunit, which associates with the ligand binding subunit of each receptor [30–32, 38]. Members of the Src family kinase, such as Hck and Lyn, are necessary for tyrosine phosphorylation of the ITAM motif in FcR*γ* [33, 39]. Tyrosine phosphorylated ITAM serves as a docking site for Syk, a member of the Syk protein kinase family, and the phosphorylation of tyrosine residues within Syk occurred by Hck/Lyn as well as by autophosphorylation [34, 39–43]. Tyrosine-phosphorylated Syk is an important molecule in downstream signal transduction from Fc*γ*R activation after ligand binding and receptor aggregation [19, 39, 43]. 

We thought that when BMDC was stimulated with LPS in the presence of IVIG, Fc*γ*RI and/or Fc*γ*RIII signaling pathways were activated and both Syk and ERK1/2 were phosphorylated to increase IL-10 production. Hence, we first examined whether IVIG could induce Syk phosphorylation in BMDC stimulated with LPS. As we expected, Syk phosphorylation was observed when cells were treated with IVIG and LPS, but not with LPS alone. Next, to confirm that IVIG induced Fc*γ*R activation, we added two novel protein tyrosine kinase inhibitors, the Hck/Lyn inhibitor PP2 and Syk inhibitor piceatannol, to the culture of LPS-stimulated BMDC together with IVIG and evaluated both IL-10 production and the phosphorylation of ERK1/2 and Syk. We found that the enhancing effect of IVIG on IL-10 production diminished when cells were stimulated with LPS in the presence of PP2 and piceatannol. Furthermore, the increase in the phosphorylation of ERK1/2 and Syk by IVIG after stimulation with LPS was reduced by the treatment with PP2 and piceatannol. These results suggest that Fc*γ*R activation is an important factor in the enhancing effect of IVIG on the production of IL-10, and Src family kinases, such as Hck/Lyn, and the Syk family kinase, Syk, are together involved in the regulation of cytokine production in LPS-stimulated BMDC in the presence of  IVIG. 

It is interesting that the increase in IL-10 production by IVIG disappeared when LPS-stimulated BMDCs were cultured with IVIG and the anti-Fc*γ*RI antibody, whereas a decreasing effect on IL-12p70 production was not affected by the antibody in the same culture conditions. These findings indicate that at least Fc*γ*RI, one of two Fc*γ*Rs involved in immune activation, mediates the upregulation of IL-10 production by IVIG, but the same receptor seems to have had no suppressing effect on IL-12p70 production. The mechanism of these functional differences is not clear, but we think one possibility that Fc*γ*RI and Fc*γ*RIII (if any, Fc*γ*RIIB) together contribute to the hyperproduction of IL-10 of BMDC stimulated with LPS in the presence of IVIG. As this production of IL-10 by IVIG in LPS-stimulated BMDC was sensitive to inhibitors of Hck/Lyn and Syk (both kinases are necessary to Fc*γ*RI and Fc*γ*RIII signal transduction), we thought that activation of an Fc*γ*RIII-mediated signaling would occur by IVIG with LPS and the signal from this receptor could induce IL-10 production likely in the case of Fc*γ*RI. When we added the anti-Fc*γ*RI antibody in the culture of LPS-stimulated BMDC with IVIG, IL-10 production through Fc*γ*RI activation was inhibited but Fc*γ*RIII-mediated IL-10 production might not be affected and IL-10 remaining in the medium could be enough to inhibit the IL-12 p70 production. In this regard, Lories et al. reported that anti-Fc*γ*RI and anti-Fc*γ*RIII antibodies inhibited IL-10 production in human DC cultured with IVIG [44]. 

In contrast to the absence of an effect by the anti-Fc*γ*RI antibody on the suppressing activity of IVIG on the production of IL-12p70, the anti-IL-10 antibody treatment clearly restored the IL-12p70 decrease by IVIG. All isotype antibodies used in these experiments had no influence on the cytokine production in BMDC stimulated with LPS. These findings suggest that the IL-10 hyperproduced by IVIG through Fc*γ*RI activation is important for the suppression of IL-12p70 production. However, the possibility of Fc*γ*RIII-mediated signal transduction and the contribution of inhibitory Fc*γ*RIIB should be also considered to understand the effects of IVIG on the production of IL-10 and IL-12p70 more clearly. As our preliminary study using an antibody that binds Fc*γ*RIIB and Fc*γ*RIII showed an inhibitory effect on IL-10 production by IVIG in cells stimulated with LPS (data not shown), we thought that receptors other than Fc*γ*RI, Fc*γ*RIII, and Fc*γ*RIIB may play important roles in the modulatory functions of IVIG. Furthermore, whether Fc*γ*RIIB could exert its inhibitory effects on signal transduction of TLR4 and cytokine production in BMDC induced by Fc*γ*R activation needs to be investigated. 

The functionally important structures of IVIG in its immunomodulatory function were not determined in this study. Without LPS stimulation, IVIG showed no influence on cytokine production in BMDC in our study. As IVIG contains an LPS-binding fraction, it seems that IVIG could exert immunomodulatory effects on cells through an immune complex with LPS. LPS is a possible partner in the formation of an immune complex with IVIG, although further studies are required to confirm this [16]. In our preliminary experiments, neither Fab nor F(ab′)_2_ fragments increased IL-10 production in LPS-stimulated BMDC (data not shown). We suppose that the binding activities of Fab and F(ab′)_2_ fragments against antigens in LPS were not enough for IVIG to express its modulatory effects on the production of the cytokine and a help of Fc region might be required for the immunoregulatory function of IVIG. 

In contrast to the mechanisms of the enhancing effect of IVIG on the production of IL-10 in BMDC stimulated with LPS, those of the suppressing effect on IL-12p70 production by IVIG are thought to be more controversial. There may be at least two possibilities for the mechanisms of the suppressing effect of IVIG on the production of IL-12p70 in the cells stimulated with LPS. One is LPS-binding neutralizing antibodies in IVIG [16]. Signals required for IL-12p70 production may be inhibited by blocking LPS-induced activation in target cells. We observed that p38 MAPK phosphorylation was reduced in LPS-stimulated BMDC in the presence of IVIG. p38 MAPK and JNK activation has been reported to be necessary for the production of IL-12p70 by DC after stimulation with LPS [26, 27]. We first thought that IL-10 had a possibility to reduce the phosphorylation of p38 MAPK. But if IL-10 could affect p38 MAPK phosphorylation, the production of this cytokine was seemed too early (within 15 min after stimulation) and considerably small amount in quantity (seems about several pg/mL) to show its reducing effect. Furthermore, Donnelly and coworkers reported that IL-10 showed no clear inhibitory effect on the phosphorylation of p38 MAPK of human monocyte stimulated with LPS *in vitro* [45]. In their report, purified human monocytes were pretreated with IL-10 (10 ng/mL) for 1 hour and were incubated with LPS (100 ng/mL) for 0 to 60 min and they found that pretreatment with IL-10 did not decrease the levels of phospho-p38 MAPK. We thought that IL-10 might not affect p38 MAPK phosphorylation in the immediate early phase after LPS stimulation and an inhibitory effect of this anti-inflammatory cytokine would appear at later phase when its production increased. Although we could not detect any clear change of JNK phosphorylation by IVIG in our study, we think that neutralization by IVIG might contribute to the reduction of phospho-p38 MAPK in the early phase (within 15 min after stimulation) after LPS stimulation and result in the suppression of IL-12p70 production. Another possibility was the unidirectional negative regulation of IL-12p70 by IL-10 in LPS-activated DC as reported by Kao [5]. They demonstrated the reverse relationship between the production of IL-10 and IL-12p70 in activated DC. In their study, it was found that exogenous IL-10 suppressed the production of IL-12p70, while the addition of IL-12p70 did not suppress IL-10 production in LPS-activated DC [5]. They also reported that an ERK inhibitor significantly suppressed IL-10 and increased IL-12p70 production. It is likely that IL-10 and the IL-10 receptor-mediated signaling pathway suppresses the production of IL-12p70 in DC. In our study, when cells were stimulated with LPS, IVIG induced the upregulation of ERK1/2 phosphorylation by activation of Fc*γ*R-mediated mechanism and this led to an increase in IL-10 production. Increased IL-10 could suppress IL-12p70 production by its negative regulation on cytokine production and may maintain DC in an immature state to exert immunosuppressive functions. These putative mechanisms of modulatory effect of IVIG on cytokine production were illustrated in in Supplement data Figure S3.

Signal transduction through Fc*γ*RI activation by the immune complex of IVIG and LPS is one possible mechanism to explain this enhancing effect of IVIG on the production of IL-10 in BMDC stimulated with LPS. However, a clear mechanism including Fc*γ*RIIB and Fc*γ*RIII for the effects of IVIG on IL-10 and L-12p70 production remains to be determined, and further studies are necessary to demonstrate that the same mechanism works in human DC stimulated with LPS. 

In conclusion, the results of this study clearly showed that IVIG had an enhancing effect on IL-10 production in mouse BMDC stimulated with LPS through Fc*γ*R activation. The suppression of IL-12p70 production by IVIG depends on IL-10 secreted, and inhibition of the LPS-TLR4 signaling pathway may also contribute to the suppressive effect of IVIG. In conjunction with inhibitory Fc*γ*RIIB in mouse DC, signals from activated Fc*γ*R may be a novel mechanism through which IVIG expresses its immunosuppressing functions.

## Supplementary Material

This Supplement data includes two experimental results (Figure S1 and Figure S2) which we mentioned in our report and one illustration (Figure S3) to help with the understanding of the readers about the mechanism studied in the report. Figure S1 shows effects of IVIG on the production of IL-10 and IL-12 p70 of BMDC stimulated with LPS for 6 h. Effects of IVIG on the mRNA transcription of IL-10 and IL-12 p70 of LPS-stimulated cells for 18 h were shown in Figure S2. Figure S3 illustrates the putative targets and mechanisms of IVIG on the cytokine production of BMDC.

## Abbreviations

BMDC: Bone-marrow-derived dendritic cells
Fc*γ*R: Fc gamma receptor
FCS: Fetal calf serum
rmGM-CSF: Recombinant murine granulocyte-macrophage colony-stimulating factor
IL-10: Interleukin 10
IL-12p70: Interleukin 12p70
IVIG: Intravenous immunoglobulin
LPS: Lipopolysaccharide
TLR4: Toll-like receptor 4.
