# Supplementary material for: An Fc Gamma Receptor-Mediated Upregulation of the Production of Interleukin 10 by Intravenous Immunoglobulin in Bone-Marrow-Derived Mouse Dendritic Cells Stimulated with Lipopolysaccharide In Vitro
Source: J Signal Transduct. 2013 Jun 18;2013:239320. doi: 10.1155/2013/239320 (PMC3703884; doi:10.1155/2013/239320)
Supplement: Supplementary file 1 — This Supplement data includes two experimental results (Figure S1 and Figure S2) which we mentioned in our report and one illustration (Figure S3) to help with the understanding of the readers about the mechanism studied in the report. Figure S1 shows effects of IVIG on the production of IL-10 and IL-12 p70 of BMDC stimulated with LPS for 6 h. Effects of IVIG on the mRNA transcription of IL-10 and IL-12 p70 of LPS-stimulated cells for 18 h were shown in Figure S2. Figure S3 illustrates the putative targets and mechanisms of IVIG on the cytokine production of BMDC. [file 239320.f1.pdf]

Supplement Data

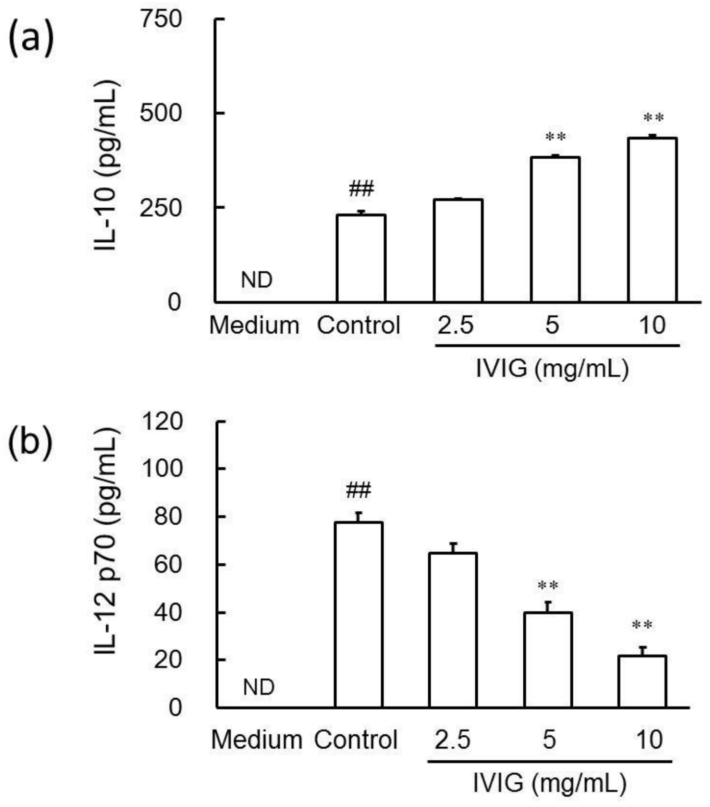


Figure S1: IVIG increased IL-10 production whereas decreased IL-12p70 production in BMDC stimulated with LPS.

Cells were stimulated with LPS (1 μg/mL) in the presence of IVIG for 6 h. Cytokine concentrations in the culture medium were determined using respective ELISA kits. (a) Production of IL-10; (b) Production of IL-12p70. Results were expressed as mean±SEM (n=3). $^{\#\#}p<0.01$, significantly different from the medium alone (without LPS stimulation, Student's t-test); $^{**}p<0.01$, significantly different from the Control (LPS stimulation without IVIG, Dunnett's multiple comparison test); ND, IL-10 concentrations were under the lower limit values of the standard ELISA curves. At least three independent experiments were conducted and representative results were shown.

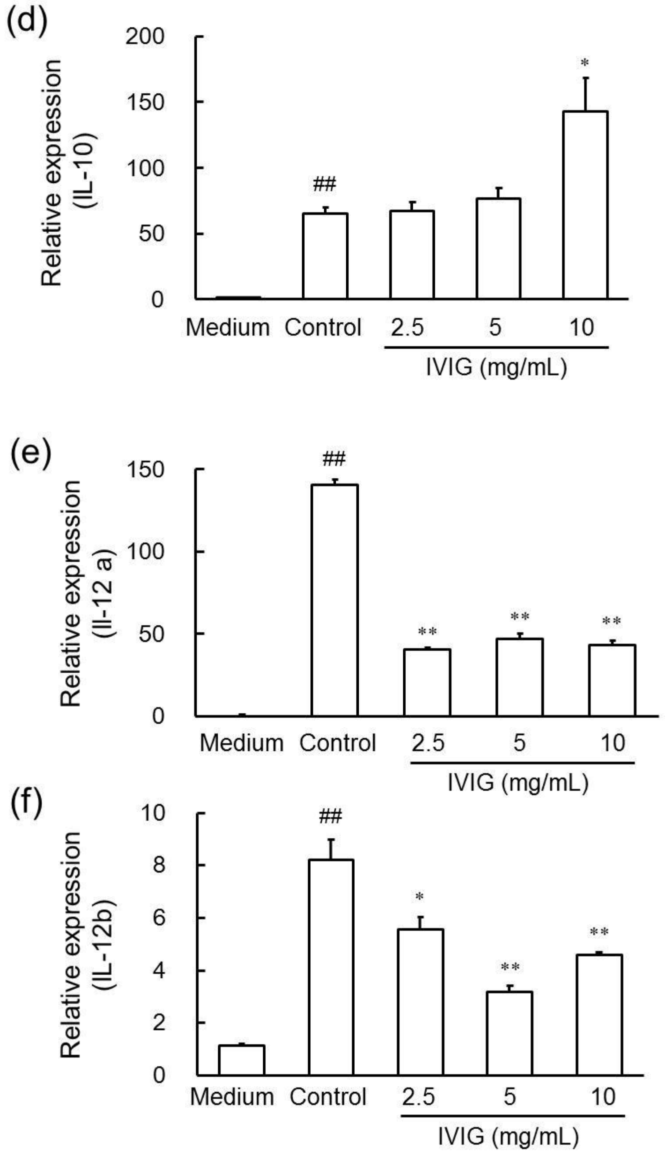


Figure S2: IVIG increased IL-10 mRNA transcription whereas decreased IL-12 mRNA transcription in BMDC stimulated with LPS.

Cells were stimulated with LPS (1 μg/mL) in the presence of IVIG for 18 h. The expressions of mRNA for IL-10, IL-12a, and IL-12b were determined by real-time quantitative RT-PCR. Results were expressed as mean±SEM (n=3). $^{\#\#}p<0.01$, significantly different from the medium alone (without LPS stimulation, Student's t-test); $^{*}p<0.05$, $^{**}p<0.01$, significantly different from the Control (LPS stimulation without IVIG, Dunnett's multiple comparison test). At least three independent experiments were conducted and representative results were shown. (a) Expression of IL-10; (b) Expression of IL-12a; (c) Expression of IL-12b.

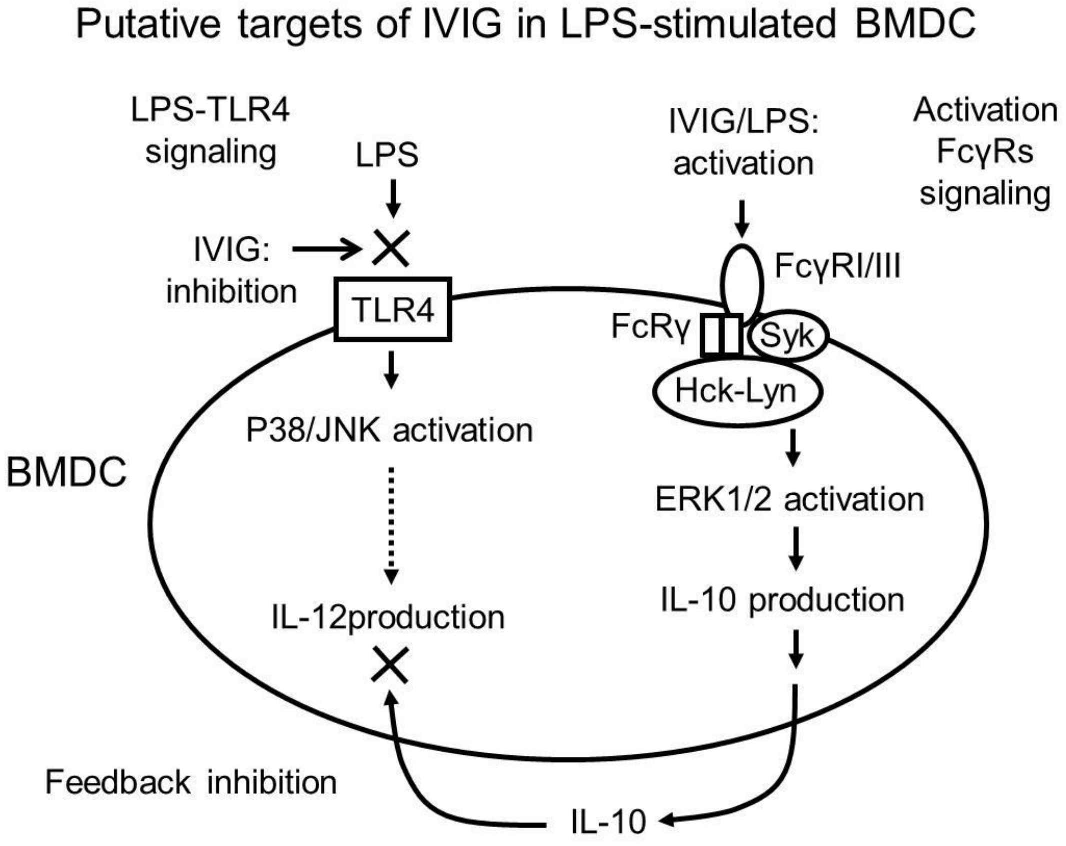


Figure S3: A putative mechanism of IVIG in LPS-stimulated BMDC

(a) After stimulation BMDC with LPS, IVIG inhibits TLR4 signal transduction and reduces p38 MAPK phosphorylation. This may lead to the inhibition of IL-12p70 production. (b) IVIG/LPS complex activates FcγR signaling and induces Syk phosphorylation and ERK1/2 hyper-phosphorylation. This activation results in the up-regulation of IL-10 production and the increased IL-10 contributes to the down-regulation of IL-12p70 production.
